# Ein Fahrradsturz mit Folgen

**DOI:** 10.1007/s00120-023-02202-5

**Published:** 2023-10-10

**Authors:** Wilhelm Schreen, B. Huch, D. Vogele, F. Zengerling

**Affiliations:** 1 Klinik für Urologie- und Kinderurologie, Universitätsklinikum Ulm, Oberer Eselsberg 23, 89081 Ulm, Deutschland; 2 Klinik für Diagnostische und Interventionelle Radiologie, Universitätsklinikum Ulm, Oberer Eselsberg 23, 89081 Ulm, Deutschland

## Anamnese

Ein 24-jähriger Patient stellte sich nach stattgehabtem Sturz vom Fahrrad in unserer interdisziplinären Notaufnahme vor. Der Patient berichtete über einen Aufprall der Peniswurzel gegen den Fahrradlenker im Rahmen des Sturzes. Initial seien rechtsseitige Schmerzen im Skrotum und eine leichte, spontan sistierende urethrale Blutung bei jedoch ungestörter Miktion aufgetreten. Bezüglich Vorerkrankungen berichtete der Patient über ein bekanntes Von-Willebrand-Jürgens-Syndrom Typ IIA (Subtyp IIE). Nach unauffälliger klinischer und sonographischer Untersuchung wurde er mit oraler Analgesie in die ambulante Weiterbehandlung entlassen.

Vier Tage später stellte sich der Patient mit einer inkompletten Tumeszenz des Penis wieder vor. Die Erektion bestand anamnestisch seit 48 h ohne neuerliche Makrohämaturie.

## Klinischer Befund und Bildgebung

Bei der körperlichen Untersuchung fand sich inspektorisch eine nicht druckschmerzhafte, im Bereich der rechten Penisbasis befindliche Resistenz im Sinne eines Hämatoms. Ansonsten zeigten sich Penis sowie Hoden nicht tastsuspekt.

**Figure 1:**
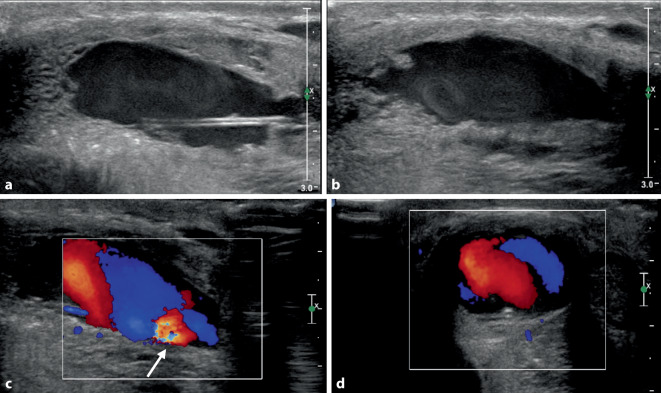

Anschließend erfolgte die Sonographie des äußeren Genitals. Hier zeigte sich im B‑Bild eine ca. 34 × 23 × 15 mm messende, echoarme ovaläre Struktur als Zeichen einer mutmaßlichen Shunthöhle bei arteriocavernöser Fistel im Corpus cavernosum um die rechte A. penis profunda (Abb. 1a). Schon im B‑Bild waren innerhalb der Höhle echoreiche „Verwirbelungen“ nachweisbar (Abb. 1b), wie bei einem arteriellen Jet im Sinne eines High-flow-Priapismus. Die farbkodierte Duplexsonographie (FKDS) zeigte ein entsprechend starkes pulsatiles Flusssignal innerhalb der Shunthöhle (Abb. 1c, d).

Eine BGA wurde leider nicht durchgeführt.

**Figure 2:**
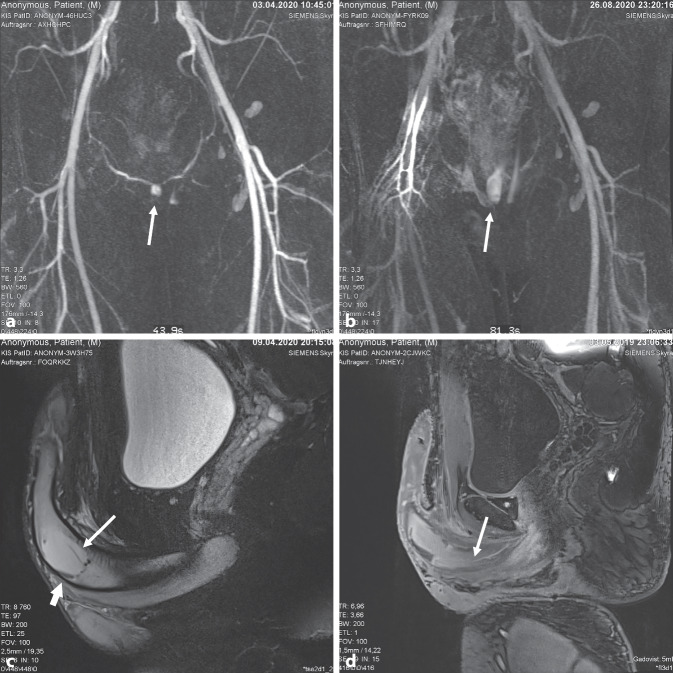

Zur weiteren Diagnostik und Therapieplanung erfolgte eine (Angio‑)MRT des Beckens mit Kontrastmittel. In Korrelation zur Sonographie stellte sich ein arteriovenöser Shunt (Abb. 2a, b) im rechtsseitigen Corpus cavernosum am Übergang der Penisbasis zum Penisschaft ausgehend von der rechtsseitigen A. penis profunda mit der bekannten Shunthöhle (Abb. 2c) und einem KM-Abbruch der Arterie dar (Abb. 2d). Hinweise auf eine Verletzung der Urethra fanden sich nicht.

## Wie lautet Ihre Diagnose?

## Therapie und Verlauf

Im interdisziplinären Konsens wurde die Indikation zur Embolisation der bestehenden Fistel gestellt. Neben der entsprechenden Aufklärung erfolgte eine gerinnungsstabilisierende Vorbereitung bei einer Von-Willebrand-Faktor (vWF)-Aktivität von 11 %. Dies beinhaltete die Substitution von 4000 i. E. Faktor VIII sowie 9600 i. E. vWF.

**Figure 3:**
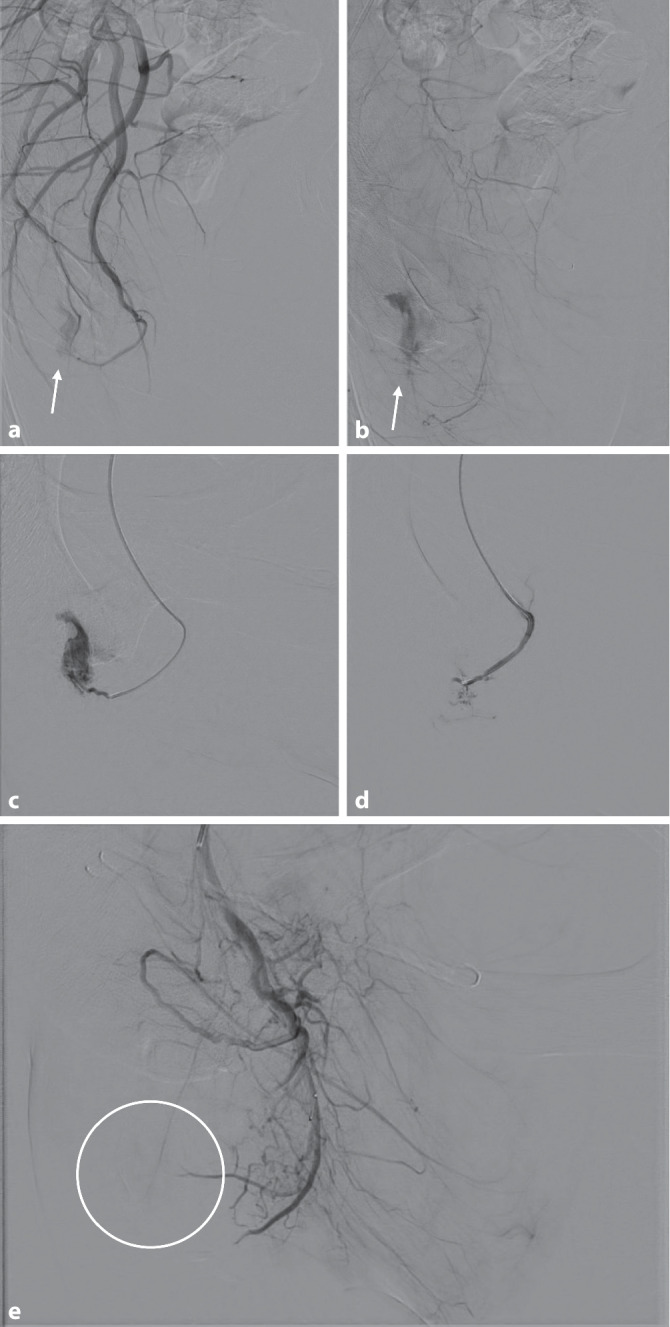

Die Abb. 3 zeigt die Bilder der transarteriellen Intervention via digitaler Subtraktionsangiographie (DSA). Zunächst erfolgte die Darstellung über einen Katheter in der rechten A. iliaca interna (Abb. 3a). Mit einem 2,4-French-Mikrokatheter wurden die arteriellen Feeder des AV-Shunts aus kleinen Ästen der A. pudenda interna rechts sondiert und selektiv dargestellt (Abb. 3c). Die superselektive Embolisation erfolgte mittels Eigenblutkoagulation. Bei kleiner periinterventioneller Blutung aus einem arteriellen Ast wurde zusätzlich eine Coil-Embolisation durchgeführt. In der Kontroll-DSA war der AV-Shunt nicht mehr abgrenzbar (Abb. 3e).

Nach Entfernung des Kompressionsverbandes am ersten postinterventionellen Tag zeigte sich die Shunthöhle sonographisch deutlich regredient. Bei klinisch stabilem, asymptomatischem Zustand erfolgte die Entlassung des Patienten nach Hause.

Zirka 14 Tage später präsentierte er sich erneut mit subjektiv zunehmender Schwellung im Bereich der ehemaligen AV-Fistel ohne Schmerzsymptomatik oder Makrohämaturie. Sonographisch zeigte sich erneut eine 12 × 5 × 5 mm messende Shunthöhle mit entsprechendem Flusssignal in der FKDS.

**Figure 4:**
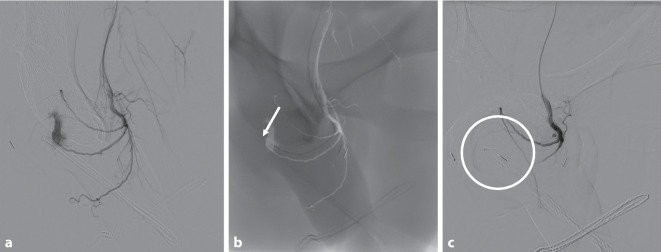

Zunächst wurde eine konservative Therapie mit adaptierter Spasmoanalgesie und Kompressionsverband des Penis eingeleitet. Unter dieser Therapie zeigte sich die Shunthöhle sonographisch nicht regredient, weshalb eine interventionelle Reembolisation durch die Kollegen der Radiologie durchgeführt wurde (Abb. 4). Hierbei erfolgte die erfolgreiche superselektive Embolisation einer kleinen weiteren Feeder-Arterie aus der Pudenda interna mittels „fiber coil“ (5 mm). In den folgenden sonographischen Kontrollen zeigte sich die Resthöhle ca. 10 × 2 × 2 mm messend ohne relevante Vaskularisation in der FKDS.

## Hintergrund

Ein Priapismus ist als Erektion definiert, welche mehr als 4 h andauert. Unterteilt wird dieses Krankheitsbild in Low-flow- bzw. venookklusiven Priapismus und High-flow- bzw. arteriellen Priapismus, wobei die Low-flow-Form etwa 95 % aller Episoden ausmacht [1].

Die arterielle Form entsteht zumeist nach penilem Trauma, wobei die Energie des Traumas zu einer Verletzung des zuführenden Gefäßes im Bereich des Corpus cavernosum und somit zur Ausbildung einer Shunthöhle führt. Pathophysiologisch tritt die AV-Fistelbildung typischerweise bis zu 14 Tage nach Verletzung/Trauma, aufgrund eines Spasmus oder Ischämie der betroffenen Arterie, infolge einer unwillkürlichen oder willkürlichen Erektion auf [2].

Therapeutisch bietet sich bei diesem Krankheitsbild ein konservativer Ansatz an, welcher eine penile oder perineale Kompression beinhaltet und eine Erfolgsrate von bis zu 60 % verspricht [3]. Alternativ besteht die Möglichkeit der superselektiven Embolisation eines zuführenden arteriellen Gefäßes mittels temporären Embolisaten wie Eigenblut oder Gelatine (Gelfoam) oder permanenten Embolisaten wie Microcoils aus Metall oder Ethylen-Vinylalkohol-Kopolymeren (PVA; [4, 5]). Dabei ist beim Einsatz temporärer Embolisate ein niedrigeres Risiko einer postinterventionellen erektilen Dysfunktion beschrieben [6]. Die publizierten Erfolgsraten liegen im Intervall von 62–83 % der behandelten Patienten [7]. Diese Intervention kann bei Bedarf im Falle eines Rezidivs/insuffizienter Besserung auch wiederholt werden. In der Literatur werden als typische Komplikationen eine gluteale Ischämie, Cavernositis oder die Entstehung perinealer Abszesse beschrieben [8].

**Diagnose:** arteriovenöse Fistel im Bereich des Corpus cavernosum

In unserem Fall muss bei der therapeutischen Entscheidung die Gerinnungsstörung des Patienten mitberücksichtigt werden. Hierbei handelte es sich um ein Von-Willebrand-Jürgens-Syndrom vom Typ IIA (Subtyp IIE), welches mit einer höheren Blutungsneigung einhergeht. Diese Erkrankung ist mit ca. 1 % aller Fälle die am häufigsten auftretende hereditäre Gerinnungsstörung weltweit. Der Typ II betrifft etwa 20 % aller Patienten und wird dominant vererbt, wobei bei unserem Patienten ein Strukturdefekt vorliegt, welcher die Polymerisation der Untereinheiten des vWF verhindert [9]. Zu beachten ist deshalb vor geplanten Interventionen eine Blutungsprophylaxe mit Supplementation von Faktor VIII und vWF.

## Fazit

- Anamnestisch sollte beim Verdacht auf eine Fistelbildung besonders auf einen nichtschmerzhaften Priapismus sowie den verzögerten Beginn nach stattgehabtem Trauma geachtet werden.
- Für die Behandlung des High-flow-Priapismus kann zunächst eine konservative Therapie in Erwägung gezogen werden.
- Die supraselektive Embolisation stellt eine sichere und suffiziente Therapieoption dar.
